# Supplementary material for: Characterization of NEB pathogenic variants in patients reveals novel nemaline myopathy disease mechanisms and omecamtiv mecarbil force effects
Source: Acta Neuropathol. 2024 Apr 18;147(1):72. doi: 10.1007/s00401-024-02726-w (PMC11026289; doi:10.1007/s00401-024-02726-w)
Supplement: Supplementary file 1 — Supplementary file1 (PDF 3774 KB) [file 401_2024_2726_MOESM1_ESM.pdf]

Supplementary Figures 1-8

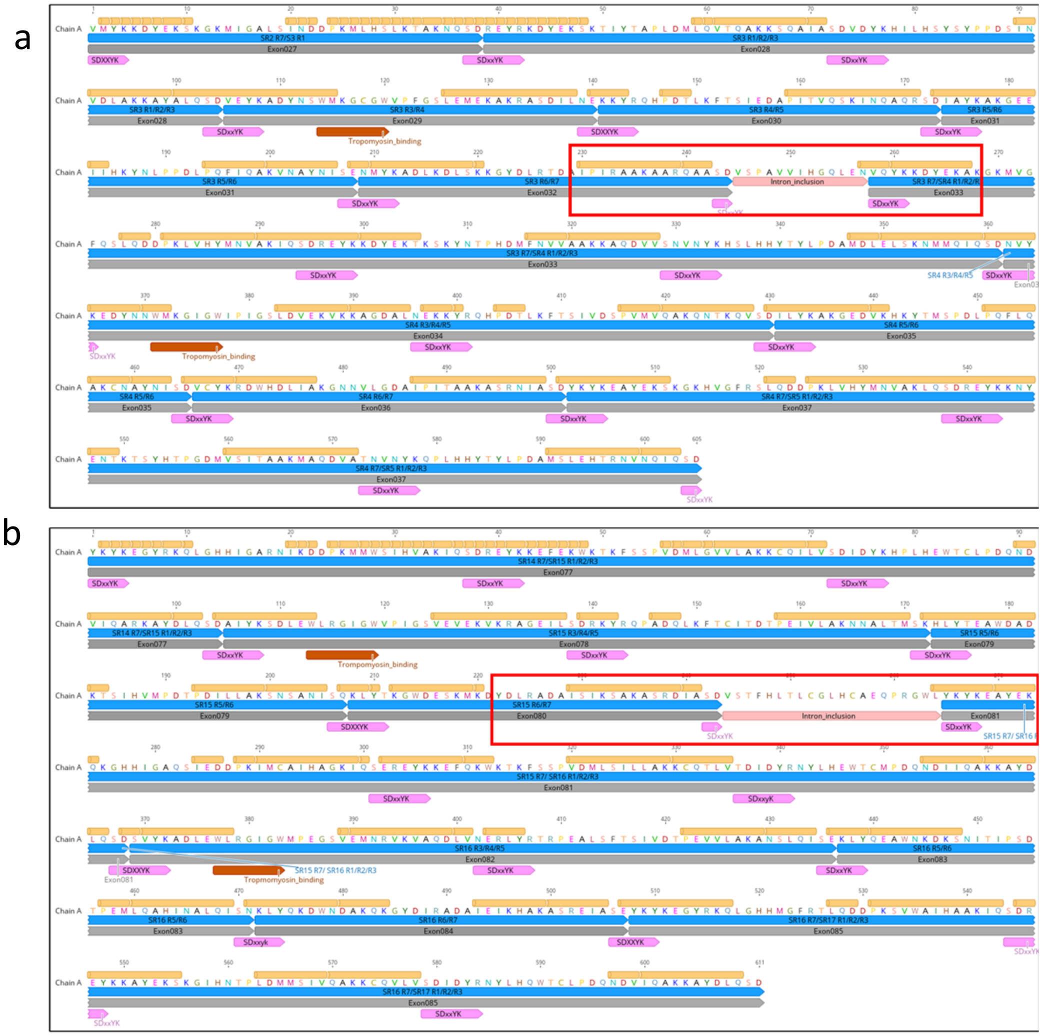

Supplementary Fig. 1 Predicted structure of mutated nebulin in patients 4001 and 2622. The regular arrangement of nebulin's simple repeats, which include actin binding sites, is interrupted by the partial inclusion of intron 32 in patient 4001 (**a**) and intron 80 in patient 2622 (**b**). Predicted nebulin α-helix structure is in yellow, super-repeat region is in blue, acting-binding motif is in pink and exon is in grey.

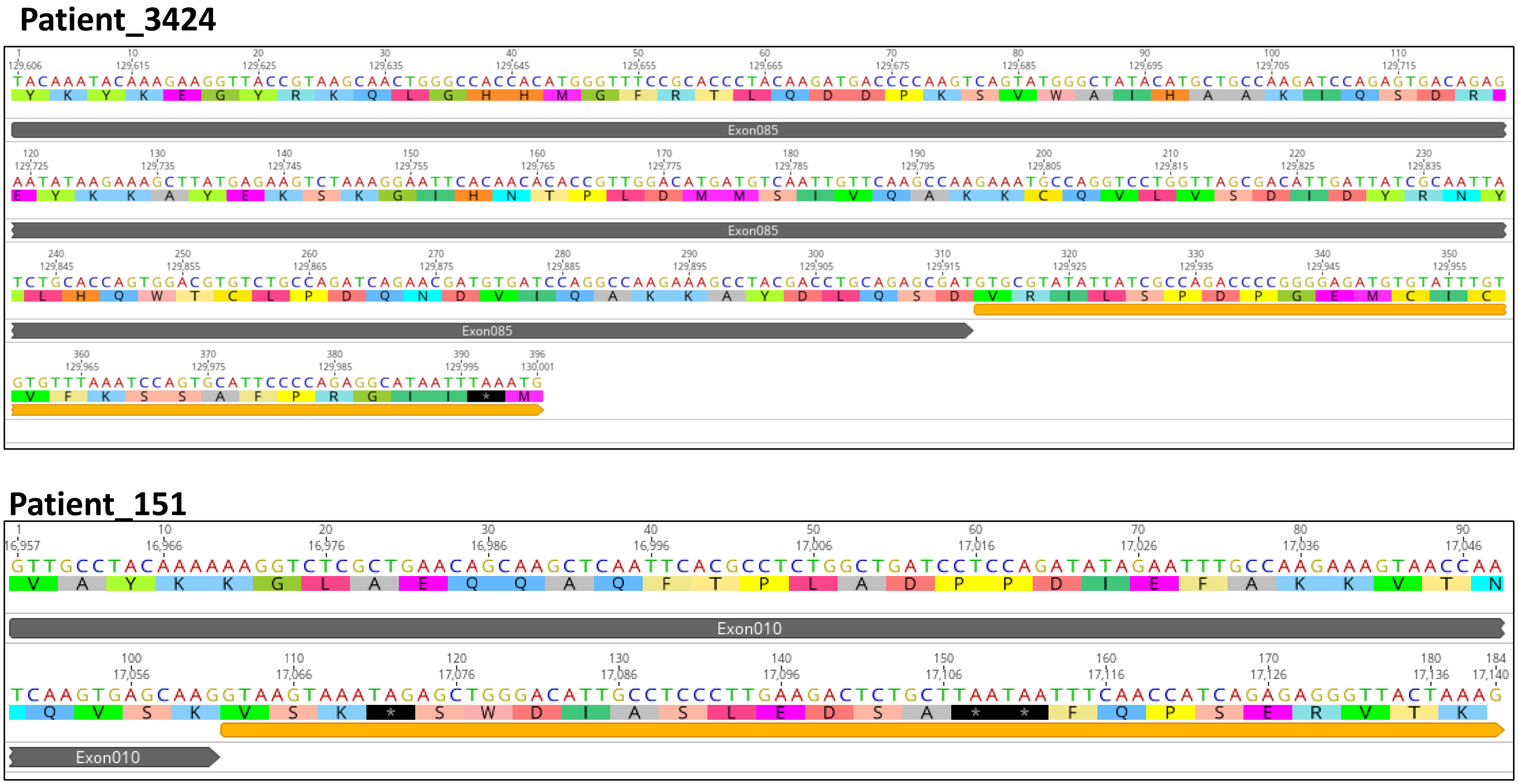

Supplementary Fig. 2 Intron inclusion outcome in patients 3424 and 151. The top panel illustrates the in-frame partial inclusion of intron 85 in patient 3424, while the bottom panel shows the out-of-frame partial inclusion of intron 10, both triggering a premature stop codon and subsequent transcript degradation through NMD.

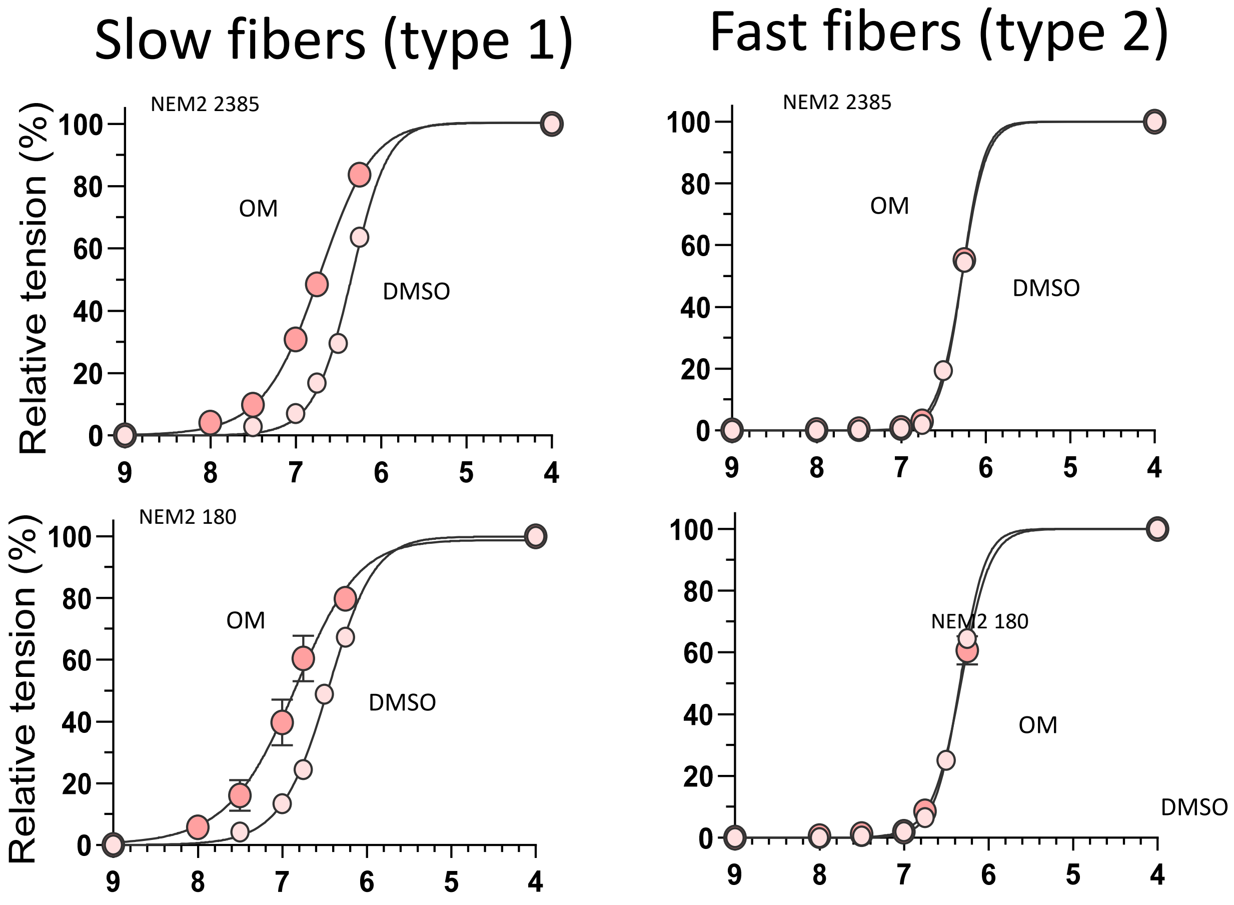

Supplementary Fig. 3 Effect of Omecamtiv mercarbil (OM) on type I (slow) and II (fast) fibers from NEM2 patients. 0.5 µM OM treatment of fast fibers (Type 2) does not affect the force-pCa relation but in slow (type 1) fibers results in a left shift.

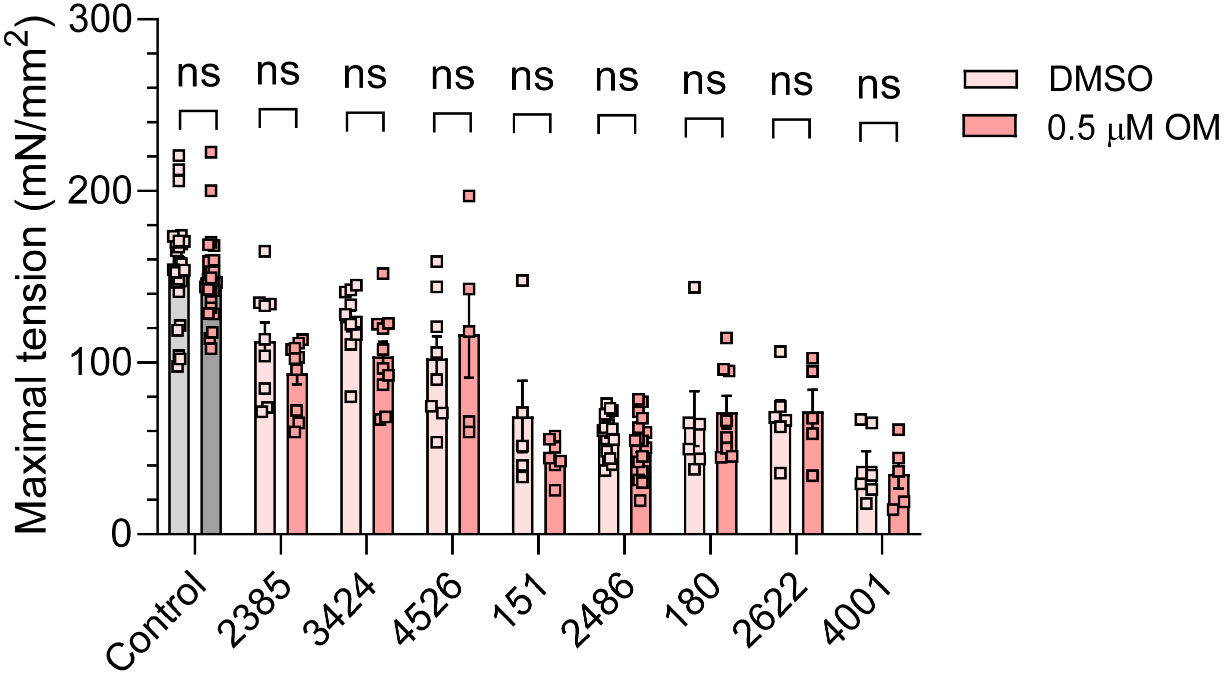

Supplementary Fig. 4 Effect of Omecamtiv mercarbil (OM) on maximal tension (pCa 4). The maximal tension of controls and NEM2 patients (regardless of nebulin level) is not changed by 0.5 μM OM treatment. ns indicates no significant difference.

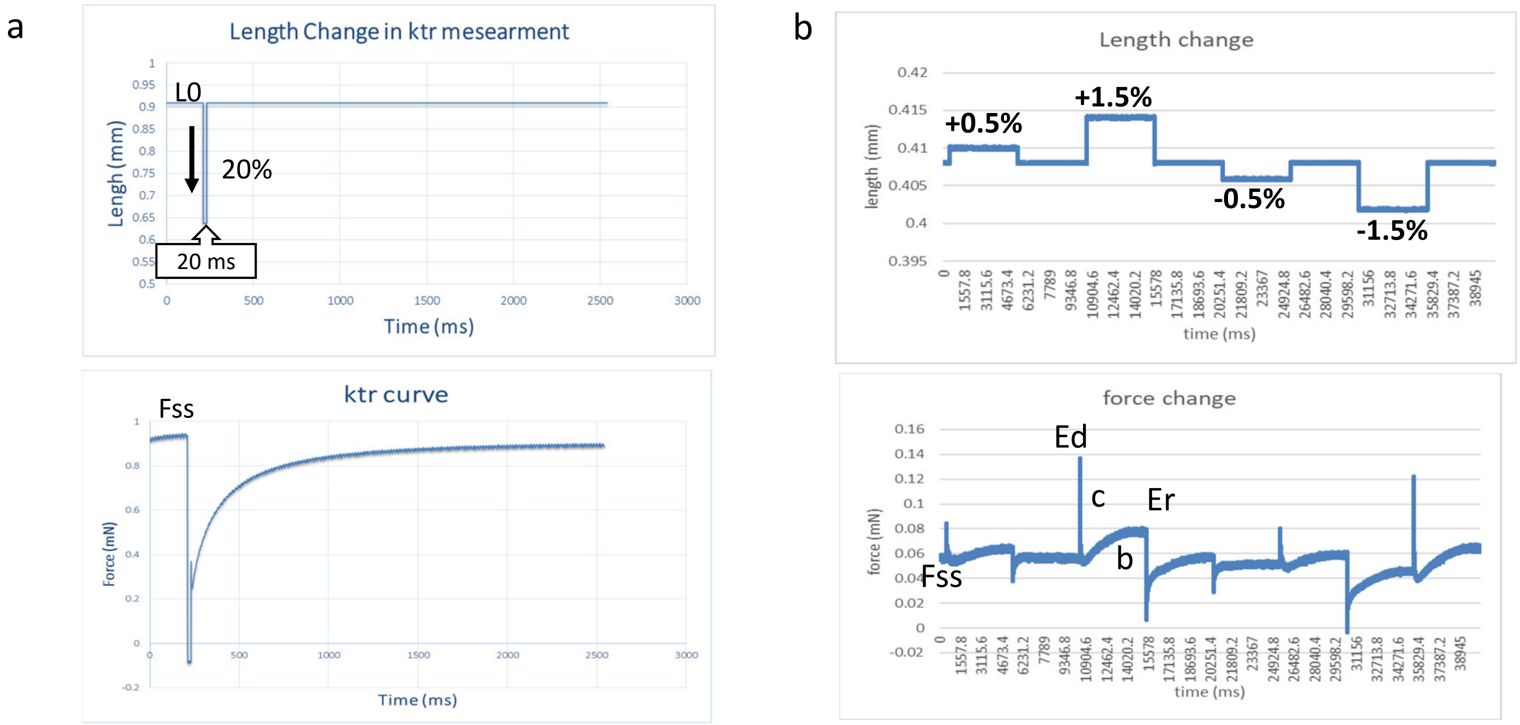

Supplementary Fig. 5 Protocol and tension transient for rate of tension redevelopment (*ktr*) and dynamic stiffness measurements. a Fiber length change (top) and force trace (bottom) for measuring ktr. At steady-state force (Fss) the muscle fiber was rapidly (<1 ms) shortened by 20% at one end of the fiber. After 20 ms the fiber was stretched to its initial length. ktr was determined by fitting the rise of force to the following equation: $F = Fss*(1-ektr*t)+c$, where F is force at time t, Fss is steady-state force. **b** Analysis of Dynamic Stiffness: At Fss, a sequence of rapid release and stretch perturbations was introduced to the fiber preparations (Top). Subsequently, the distinct phases of tension transients (Bottom) that emerged due to alterations in muscle length were individually examined to gain insights into cross-bridge dynamics. The force changes were analyzed using a non-linear recruitment-distortion (NLRD) model, yielding values for Ed (an approximation of strongly bound cross-bridges), Er (an approximation of newly formed cross-bridges), *c* (an approximation of detachment rate), and *b* (an approximation of cross-bridge attachment rate).

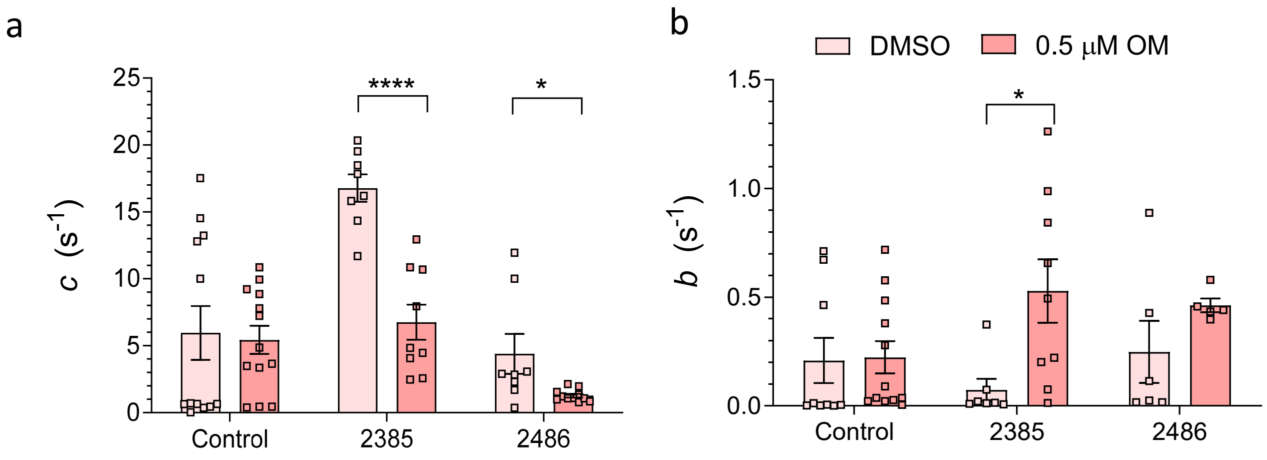

Supplementary Fig. 6 Effect of Omecamtiv mercarbil (OM) on dynamic stiffness at submaximal level of activation (pCa6.75). a. OM treatment lowers the detachment rate of cross-bridges (c) in patients but not controls. **b.** OM treatment increases attachment rate of cross-bridges (*b*) in patient 2385. Asterisks indicate a significant difference between DMSO and OM-treated fibers and ns indicates no significant difference. * *P* <0.05, ** *P* <0.01, *** *P* <0.001, **** *P* <0.0001.

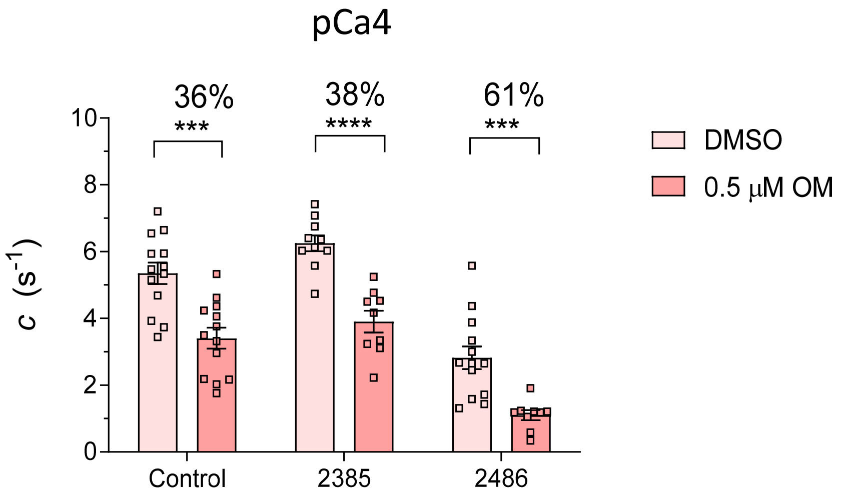

Supplementary Fig. 7. Nebulin level-dependence of OM effect on Cross-Bridge Detachment Rate (*c*). OM-treatment lowers c in the patient with low nebulin (2486) to a greater extent (61%) than the patient with normal nebulin (2385) or control (38 and 36% respectively).

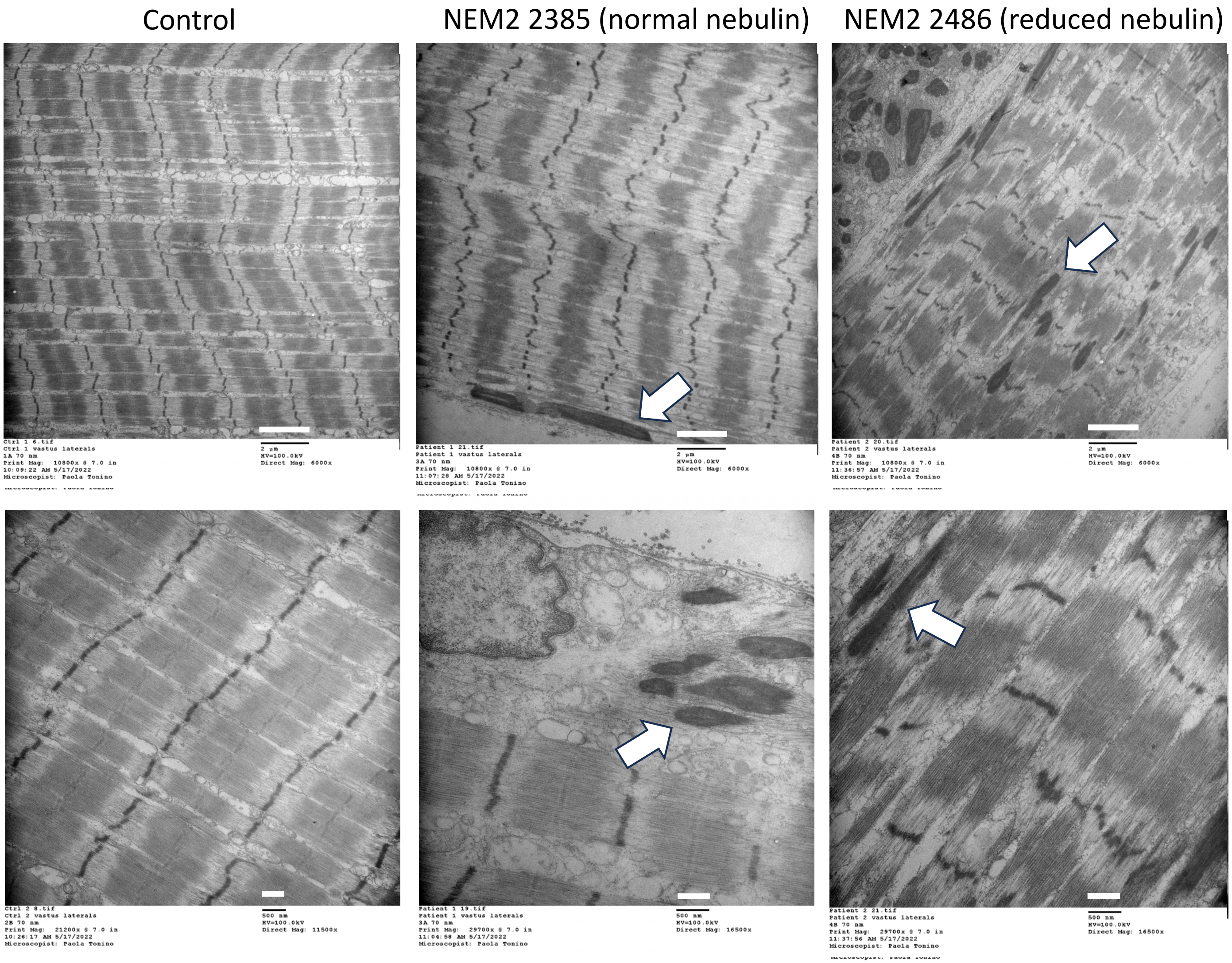

Supplementary Fig. 8. Structural studies of sarcomeric structure by electron microscopy. Low magnification (scale bar: 2 μm) (top) and high magnification (scale bar 500 nm) view of fiber bundles in control, NEM2 2385 (normal nebulin) and NEM2 2486 (reduced nebulin) muscle biopsy samples. Protein aggregates (white arrows) and misaligned sarcomeres observed frequently in patients but not in control.
