# Supplementary material for: Characterization of NEB pathogenic variants in patients reveals novel nemaline myopathy disease mechanisms and omecamtiv mecarbil force effects
Source: Acta Neuropathol. 2024 Apr 18;147(1):72. doi: 10.1007/s00401-024-02726-w (PMC11026289; doi:10.1007/s00401-024-02726-w)
Supplement: Supplementary file 2 — Supplementary file2 (DOCX 103 KB) [file 401_2024_2726_MOESM2_ESM.docx]

**Supplementary Table 1. Clinical characteristics of patients with NEM2**

| **Patient ID** | **Site of biopsy** | **Age (yrs) at biopsy** | **Gender** | **Jump both feet leaving ground** | **Walk unassisted** | **Sitting unassisted** | **Breathing tube and vent in neonatal period** | **Feeding difficulties** | **Assisted breathing** |
| --- | --- | --- | --- | --- | --- | --- | --- | --- | --- |
| 2385 | Paraspinal | 22 | F | Never able | Yes, greater than 10 steps | Unassisted more than an hour | No | Has or had G-tube & poor weight gain | Yes, > 12 hours/day |
| 2296 | paraspinal | 11 | F | Never able | Never able | Unassisted more than an hour | No | Has or had poor weight gain | Yes, > 12 hours/day |
| 2486 | Paraspinal | 5.75 | F | Never able | Never able | Unassisted more than an hour | No | Has or had G-tube & poor weight gain | Yes, > 12 hours/day |
| 3424 | right thigh | 7 | F | Yes | Yes, greater than 10 steps | Unassisted more than an hour | No | None | Yes, < 12 hours/day |
| 4001 | right thigh | 0.3 | M | Never able | Never able | Unassisted more than an hour | No | Has or had G-tube & poor weight gain | Yes, > 12 hours/day |
| 2622 | quadriceps | 0.75 | F | Never able | Never able | Unassisted more than an hour | No | Has or had NG tube | Yes, < 12 hours/day |
| 4526 | Left thigh | 2 | M | Never able | Never able | Unassisted more than an hour | No | Has or had G-tube & poor weight gain | Yes, < 12 hours/day |
| 144.0 | left tricep | 1.3 | F | Never able | Never able | Unassisted more than an hour | No | Has or had G-tube & poor weight gain | Yes, < 12 hours/day |
| 180.0 | left quad | 0.66 | F | Never able | Yes, greater than 10 steps | Unassisted more than an hour | No | Has or had G-tube & poor weight gain | Yes, > 12 hours/day |
| 151.0 | left thigh | 1 | F | Never able | Never able | Unassisted more than an hour | No | Has or had G-tube & poor weight gain | None |

**Supplementary Table 2. Summary of mutation analysis in NEM2 patients**

| **Patient** | **Mutation** | **Mutation Type** | **Isoforms of transcripts ^a^** | **Transcript (%) in patients** | **Transcript (%) in controls** | **Cryptic splice site activation** | **Allelic imbalance** | **Intronic inclusion** | **Mutation site** | **PSI (%)^b^** | **Clinical significance (ClinVar)** | **Total *NEB* transcript** | **Protein level** |
| --- | --- | --- | --- | --- | --- | --- | --- | --- | --- | --- | --- | --- | --- |
| 2385  Allele 1 | exon 32 c.3252_3255+3delTGACGTA:  Results 7 bp deletion | Deletion | 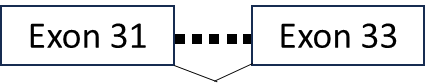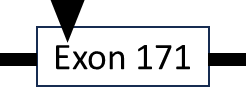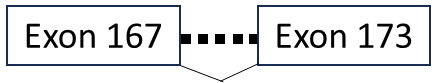 | 17.02 | 0.01 |  |  |  | SR3 R6,R7 | 82 | Likely pathogenic | Normal | Normal |
|  |  |  | 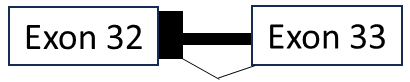 | 9.73 | 0.02 | Yes |  | Out-frame inclusion of 39 bp from intron 32 equates to addition of 13 amino acids |  |  |  |  |  |
| 2385  Allele 2 | 2.7 kb deletion exon 77 g.152,469,299 in intron 77 g.152,471,995 in intron 76:  Results 2697 deletion including exon 77 | Deletion | 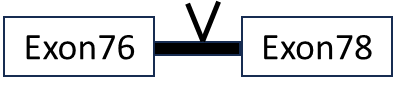 | 49.6 | 0.03 |  |  |  | SR14 R7 / SR15 R1,R2,R3 | 46 | - |  |  |
| 2296  Allele 1 | exon 112 c.17654G>A:  Results generation of stop codon in exon 112 | Truncation | 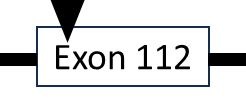 | 27 |  |  | Yes |  | SR24 R2/R3 | 100 | Pathogenic | Reduced | Reduced |
| 2296  Allele 2 | exon 175 c.24771delT:  Results deletion in exon 175 leading to stop 19 bp into exon 176 | Frameshift | 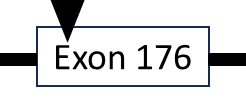 | 39.34 |  |  |  |  | M234/M235 | 100 | Pathogenic/  Likely pathogenic |  |  |
| 2486  Allele 1 | exon 61 c.8425 C>T :  Results generation of stop codon in exon 61 | Truncation | 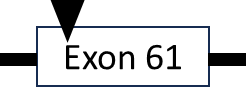 | 34 |  |  | Yes |  | SR10 R7 / SR11 R1,R2,R3 | 100 | Pathogenic/  Likely pathogenic  in LOVD | Reduced | Reduced |
| 2486  Allele 2 | exon 171 c.24317 T>A:  Results generation of stop codon in exon 171 | Truncation | 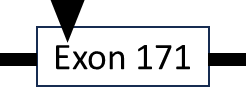 | 35 |  |  | Yes |  | M230/M231 | 77 | Pathogenic/  Likely pathogenic  in LOVD |  |  |
|  |  |  | 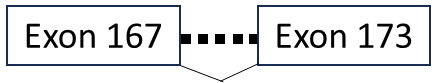 | 30.5 | 22.8 |  |  |  |  |  |  |  |  |

| **Patient** | **Mutation** | **Mutation Type** | **Isoforms of transcripts ^a^** | **Transcript (%) in patients** | **Transcript (%) in controls** | **Cryptic splice site activation** | **Allelic imbalance** | **Intronic inclusion** | **Mutation site** | **PSI (%)^b^** | **Clinical significance** | **Total NEB transcript** | **Protein level** |
| --- | --- | --- | --- | --- | --- | --- | --- | --- | --- | --- | --- | --- | --- |
| 3424  Allele 1 | exon 85 c.13059+5G>A: Results point mutation in intron 85 | Intronic point mutation | 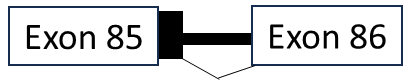 | 20.38 | 0.03 | Yes |  | In-frame intron inclusion of 84 bp from intron 85 equates an addition of 28 amino acids. The inclusion would add a stop codon before exon 86. | SR16 R7 / SR17 R1,R2,R3 | 100 | Likely pathogenic | Reduced | Normal |
| 3424  Allele 2 | exon 169 c.24218C>A: Results generation of stop codon in last codon of exon 169 | Truncation | 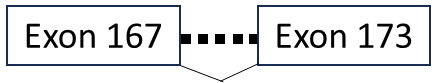 | 74.5 | 44.28 |  | Yes |  | M228/M229 (Z-disk) | 31 | VUS/  pathogenic in LOVD |  |  |
|  |  |  | 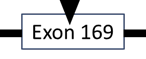 | 25.42 | 55.72 |  |  |  |  |  |  |  |  |
| 4001  Allele 1 | exon 32 c.3255+1G>A: Results donor splice mutation in junction of exon-intron 32 | Splicing | 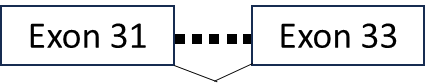 | 12.55 | 0.01 |  |  |  | SR3 R6/R7 | 85 | Pathogenic | Normal | Reduced |
|  |  |  | 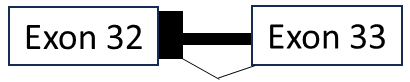 | 51.82 | 0.02 | Yes |  | In-frame inclusion of 39 bp from intron 32 equates to an additional 13 amino acids |  |  |  |  |  |
| 4001  Allele 2 | exon 110 c.17501_17502delinsC: Results insertion in exon 110 leading to stop codon 19 bp into exon 111 | Frameshift | 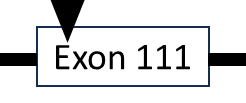 | 22.18 |  |  |  |  | SR23 R7/SR24 R1 | 100 | Pathogenic in LOVD |  |  |
| 4526  Allele 1 | exon 109 c.17262G>A:Results generation of stop codon in the middile of exon 109 | Truncation | 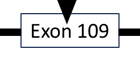 | 23.9 |  |  | Yes |  | SR22 R6/R7 | 100 | Pathogenic | Reduced | Normal |
| 4526  Allele 2 | exon 171 c.24318_24319insAA: Results insertion at the start of exon 171 leading to stop codon in exon 173 | Frameshift | 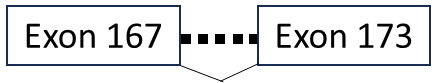 | 73.87 | 22.8 |  |  |  | M229/M230 (Z-disk) | 53 | Pathogenic |  |  |
|  |  |  | 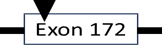 | 40 |  |  |  |  |  |  |  |  |  |

| **Patient** | **Mutation** | **Mutation Type** | **Isoforms of transcripts ^a^** | **Transcript (%) in patients** | **Transcript (%) in control** | **Cryptic splice site activation** | **Allelic imbalance** | **Intronic inclusion** | **Mutation site** | **PSI (%)^b^** | **Clinical significance** | **Total NEB transcript** | **Protein level** |
| --- | --- | --- | --- | --- | --- | --- | --- | --- | --- | --- | --- | --- | --- |
| 2622  Allele 1 | exon 80 c.12018+1G>A:  Results donor splice site mutation in juncation of exon-intron 80 | Splicing | 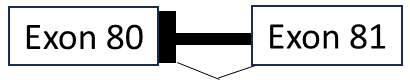 | 4.58 | 0.01 | Yes |  | In-frame inclusion of 63 bp from intron 80 equates to 21  additional amino acids | SR15 R6/R7 | 80 | Pathogenic/Likely pathogenic | Reduced | Reduced |
|  |  |  | 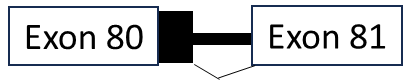 | 3.27 | 0 | Yes |  | Out-frame inclusion of 95 bp from intron 80 |  |  |  |  |  |
| 2622  Allele 2 | exon 172 c.24458_24461dupAGAT:  Duplication of AGAT in exon 172 leads to stop codon at the end of exon | Frameshift | 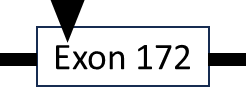 | 25 |  |  |  |  | M231/M232 | 100 | Pathogenic/Likely pathogenic |  |  |
| 144  Allele 1 & 2 | Homozygous exon 55 deletion: Results in-frame 2502 deletion including exon 55 | Deletion | 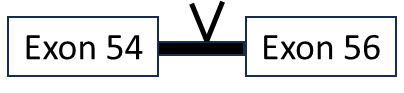 | 100 | 0 |  |  |  | SR9 R5,R6 | 4 | Pathogenic | Normal | Reduced |
| 180  Allele 2 | Four copy gain within the *NEB* gene triplicate repeat region | Duplication | 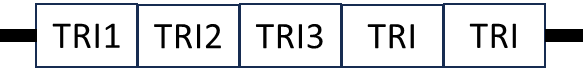 | 100 | 0 |  |  |  | [SR16 R3,R4,R5] – [SR21 R7 / SR22 R1,R2,R3] | - | Pathogenic | Reduced | Normal |
| 180  Allele 1 | exon 157 c.22936C>T (p.Arg7646*): Results generation of stop codon in exon 157 | Truncation | 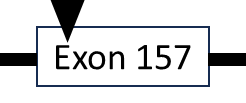 | 17 |  |  | Yes |  | M216/M217 | 100 | Pathogenic |  |  |
| 151  Allele 1 | exon 10 c.822+1G>A: Results donor splice site mutation at the junction of exon-intron 10 | Splicing | 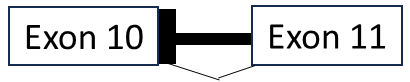 | 13.66 | 0.02 | Yes |  | Out-frame inclusion of 79 bp from intron 10. The inclusion would add 3 stop codons before exon 11. | M5 | 100 | Likely pathogenic | Reduced | Reduced |
| 151  Allele 2 | exon 30 c.3042+3_3042+6del : Results 4 bp deletion of intron 30 | Intronic Deletion | 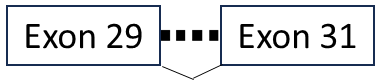 | 82.05 | 0.09 |  |  |  | R5 of SR2 | 15 | VUS |  |  |

a) Transcript isoforms are only showing isoforms are impacted by mutation and isoforms encoded by exons without mutations have not been shown.

b) PSI represents the inclusion percentage of the exons carrying mutations.

Intron , Stop codon , Exon skipping , Intron inclusion , Deleted exon


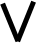

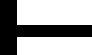

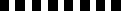

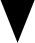

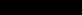


**Supplementary Table 3- Calculation of length of triplicated region in patient 180**

| **Exon NO** | **J1** | **J2** | **Reads per Exon (J1+J2)** | **Exon length** | **Normalized read count (J1+J2)/exon length** | **Normalized read count in controls** | **Ratio of normalized read counts of patient/controls** | **Exon length based on additional reads (ratio of normalized counts * exon length)** | **Added exon length in patient (exon length based on additional counts - exon length)** |
| --- | --- | --- | --- | --- | --- | --- | --- | --- | --- |
| Exon082 | 60305 | 7548 | 67853 | 203 | 334.25 | 201.53 | 1.66 | 336.69 | 133.69 |
| Exon083 | 14857 | 60279 | 75136 | 104 | 722.46 | 383.11 | 1.89 | 196.12 | 92.12 |
| Exon084 | 38236 | 14669 | 52905 | 107 | 494.44 | 247.63 | 2.00 | 213.65 | 106.65 |
| Exon085 | 36004 | 38242 | 74246 | 311 | 238.73 | 133.44 | 1.79 | 556.40 | 245.40 |
| Exon086 | 10953 | 35998 | 46951 | 203 | 231.29 | 186.95 | 1.24 | 251.15 | 48.15 |
| Exon087 | 9576 | 10902 | 20478 | 104 | 196.90 | 253.36 | 0.78 | 80.83 | -23.17 |
| Exon088 | 9582 | 9599 | 19181 | 107 | 179.26 | 203.19 | 0.88 | 94.40 | -12.60 |
| Exon089 | 43509 | 9594 | 53103 | 311 | 170.75 | 105.41 | 1.62 | 503.76 | 192.76 |
| Exon090 | 73903 | 43455 | 117358 | 203 | 578.12 | 309.80 | 1.87 | 378.81 | 175.81 |
| Exon091 | 63138 | 74076 | 137214 | 104 | 1319.37 | 744.17 | 1.77 | 184.39 | 80.39 |
| Exon092 | 57637 | 63030 | 120667 | 107 | 1127.73 | 624.17 | 1.81 | 193.33 | 86.33 |
| Exon093 | 17387 | 57609 | 74996 | 311 | 241.14 | 137.52 | 1.75 | 545.36 | 234.36 |
| Exon094 | 41034 | 17400 | 58434 | 203 | 287.85 | 123.39 | 2.33 | 473.57 | 270.57 |
| Exon095 | 34050 | 40999 | 75049 | 104 | 721.63 | 259.23 | 2.78 | 289.51 | 185.51 |
| Exon096 | 36842 | 34099 | 70941 | 107 | 663.00 | 277.89 | 2.39 | 255.28 | 148.28 |
| Exon097 | 46125 | 36846 | 82971 | 311 | 266.79 | 121.89 | 2.19 | 680.72 | 369.72 |
| Exon098 | 70513 | 46128 | 116641 | 203 | 574.59 | 302.33 | 1.90 | 385.80 | 182.80 |
| Exon099 | 66109 | 70612 | 136721 | 104 | 1314.63 | 742.93 | 1.77 | 184.03 | 80.03 |
| Exon100 | 58142 | 66109 | 124251 | 107 | 1161.22 | 647.69 | 1.79 | 191.84 | 84.84 |
| Exon101 | 16891 | 58142 | 75033 | 311 | 241.26 | 136.37 | 1.77 | 550.20 | 239.20 |
| Exon102 | 12405 | 16908 | 29313 | 203 | 144.40 | 133.31 | 1.08 | 219.89 | 16.89 |
| Exon103 | 9821 | 12405 | 22226 | 104 | 213.71 | 252.03 | 0.85 | 88.19 | -15.81 |
| Exon104 | 9710 | 9821 | 19531 | 107 | 182.53 | 196.08 | 0.93 | 99.61 | -7.39 |
| Exon105 | 8004 | 9725 | 17729 | 311 | 57.01 | 69.45 | 0.82 | 255.26 | -55.74 |
| **Additional exon based in patient 180 versus control by summing up all added exon lengths in patients** | | | | | | | | | **2858.76** |
